# A new strategy for seamless gene editing and marker recycling in *Saccharomyces cerevisiae* using lethal effect of Cwp1

**DOI:** 10.1002/mbo3.750

**Published:** 2018-10-11

**Authors:** Yuxiao Hu, Yanrong Jia, Xiangdong Zhao, Zihao Yang, Zhimin Hao, Jingao Dong, Fanli Zeng

**Affiliations:** ^1^ College of Life Sciences Hebei Agricultural University Baoding China

**Keywords:** Cwp1, gene deletion, marker recycling, *Saccharomyces cerevisiae*, seamless gene editing

## Abstract

Technologies development for seamless gene editing and marker recycling has allowed frequent genomic engineering in *Saccharomyces cerevisiae* for desired laboratory strains and cell factory. Alternative new approaches are still required for complicated scenarios. In this study, we report that inducible overexpression of cell wall protein 1 (Cwp1) by galactose addition confers yeast cells a robust growth inhibition. Direct repeats flanking the Gal‐CWP1:selectable marker cassette allow for its homology recombination excision and counter selection upon galactose addition, therefore enable seamless gene editing and marker recycling. We used this strategy and efficiently generated scarless *Ade8* deletion mutants. Our results highlight the utility of lethal effect of Cwp1 overexpression a new counter selection strategy and a simple and efficient method for seamless gene editing and marker recycling in *S. cerevisiae* and potentially other fungi.

## 1 INTRODUCTION

The budding yeast *Saccharomyces cerevisiae* is an attractive model organism for fundamental biological research and powerful cell factory for industrial application (Dikicioglu, Pir, & Oliver, 2014; Hong & Nielsen, 2012). Complex and multiple genomic engineering in *S. cerevisiae* therefore turns commonplace. However, genetic technologies innovation is still needed to enable simple and extensive genetic manipulations in such model organism.

As each modification such as gene deletion, insertion, or tagging retains one selectable marker in most current methods, it therefore presents a hurdle when dozens of genetic changes are required. Especially, industrial strains hardly modified for the use of auxotrophic selection because they are typically aneuploidy or polyploidy (Querol & Bond, 2009), thus only depends on very restricted dominant markers. Second, even if it is sufficient in use, selectable markers may have deleterious effects and interfere with physiology of host cell (Gopal, Broad, & Lloyd, 1989). Additionally, not introducing heterologous adaptors and scarless modification is always the best criterion for genomic engineering, especially for strains dedicated to food and biopharmaceutical industry.

To overcome these defects, recyclable marker and seamless genetic manipulation in *S. cerevisiae* gets rapidly developed. Both tools are getting essential in the booming field of synthetic biology and also in biotechnology industry. Several strategies for marker recycling have been in use, among which selectable markers rescue involves counter selection of auxotrophic markers is widely used for laboratory strains. Such methods include counter selection of colonies with spontaneous mutation or loss of a gene required for a specific nutrient such as *URA3*,* LYS2, CYH2,* and *MET15,* taking advantage of 5‐fluoroorotic acid (FOA), α‐aminoadipate, cycloheximide, and methyl‐mercury, respectively (Alani, Cao, & Kleckner, 1987; Brachmann et al., 1998; Chattoo et al., 1979; Käufer, Fried, Schwindinger, Jasin, & Warner, 1983; Singh & Sherman, 1974; Struhl, 1983). However, the prerequisite to use this strategy is that the original strain should be respective auxotrophic, which is hard to work for many strains, especially for prototrophic industry strains.

Another approach for marker recycling involves the use of growth inhibitory sequence such as heterologous toxin gene *mazF* derived from *Escherichia coli* (Liu et al., 2014) and yeast arrived *GIN* sequences (Akada, Hirosawa, Kawahata, Hoshida, & Nishizawa, 2002; Kawahata, Amari, Nishizawa, & Akada, 1999). Additional way to rescue the selectable marker takes advantage of site‐specific activity of a serial recombinases including Cre‐loxP system (Johansson & Hahn‐Hägerdal, 2004), FLP/FRT system (Kilby, Snaith, & Murray, 1993; Kopke, Hoff, & Kück, 2010), and I‐SceI (Solisescalante et al., 2014).

In the present study, we found that inducible overexpression of cell wall protein 1 (Cwp1) by galactose addition confers yeast cells a robust growth inhibition. We took advantage of the fact that short repeat sequences make homology recombination efficient in budding yeast and designed direct repeats flanking the Gal‐CWP1:selectable marker cassette allows for its homology recombination excision and counter selection upon galactose. We used this strategy and efficiently generated scarless *Ade8* deletion mutants validating our method for seamless gene editing and marker recycling. We therefore reported a simple and efficient method for seamless gene editing and marker recycling in *S. cerevisiae* using lethal effect of cell wall protein overexpression and hence potentially in other fungi.

## 2 MATERIALS AND METHODS

### 2.1 Strains and media

*Saccharomyces cerevisiae* parental strain used in this study is YFL3 (W3031a *ade2‐1 ura3‐1 his3‐11,15 trp1‐1 leu2‐3,112 can1‐100 bar1Δ*) that is made and preserved in our laboratory (Ren, Malik, & Zeng, 2016). Yeast cells were cultured at 30°C in rich medium with 1% yeast extract, 2% peptone, and 2% glucose (YPD media). Galactose induction medium for overexpression of Cwp1 includes 1% yeast extract, 2% peptone, and 2% glactose. For serial dilution assays, exponentially growing cultures at 30°C were spotted on the indicated plates to the same concentration using a 10‐fold serial dilution as described previously (Zhang et al., 2017).

### 2.2 Constructs generation and deletion cassette construction

pRS306‐GAL‐CWP1 and pFA6a‐GAL‐CWP1‐KanMX constructs were assembled by inserting GALl/GALl0 promoter and CWP1‐13MYC fragments into pRS306 and pFA6a‐KanMX plasmids, respectively, using AFEAP cloning method (Zeng et al., 2017).

Deletion cassettes were generated by PCR using Q5 hot start high‐fidelity DNA polymerase (NEB) and following manufacturer recommendations. Primers used for deletion cassette construction were designed as follows. The forward primers (5'–3') contain a ~55 bp sequence homologous to the region upstream the start of the fragment to delete, a followed ~55 bp sequence homologous to the region downstream the end of the fragment, and a ~20 bp sequence annealing to the upstream of plasmid template region. The reverse primers contain a sequence homologous to the last ~55 bp of the fragment to delete and a ~20 bp sequence annealing to the downstream of plasmid template region.

PCR products as the deletion cassette were transformed into the host strains using LiAc chemical transformation. For ADE8 deletion cassette, primer pairs oYX7 and oYX8 using pRS306‐GAL‐CWP1 as template and primer pairs oYX13 and oYX14 using pFA6a‐GAL‐CWP1‐KanMX as template were used, respectively. Primer pairs oZC19 and oZC20, oligo nucleotides complementary to the upstream and downstream sequences of the deletion part. The primer sequences were listed in Table 1.

**Table 1:** Oigos used in this study

Oligo	Sequence (5′−3′)
oYX7 (ade8‐URA3‐markerless‐F)	ACTTGCAGCAAGCGCAGGTGAGAGCCAACACACATCAATAATCTTTCCAAAAGCTCTCGCGTCGTAAATCATGATCATGGATTGTGACAAAACGATCTTAAAGGTTTCGAACCTTCTCTTTGGAACTTTC
oYX8 (ade8‐URA3‐markerless‐R)	ATGTTTCGCGCCTCACTTTGAAGAATGCCAAATATAAAAGTATAAATATGGGAACTATTCAGATTGTACTGAGAGTGCAC
oYX13 (ade8‐KAN‐markerless‐F)	ACTTGCAGCAAGCGCAGGTGAGAGCCAACACACATCAATAATCTTTCCAAAAGCTGAATAGTTCCCATATTTATACTTTTATATTTGGCATTCTTCAAAGTGAGGCGCGAGACATGGAGGCCCAGAATAC
oYX14 (ade8‐KAN‐markerless‐R)	TTATTTGTGAAGCTGCTGTAAAACCTTATATGTAGCTTCTACAATCGCGATGTGCTCAGCCTATAGGGAGACCGGCAGATCCGCG
oZC19 (ade8‐check‐F)	TCCAGCAAGAGGAAAGTTAT
oZC20 (ade8‐check‐R)	AGCGTTTACACATGCACATT

### 2.3 Western blotting

Whole cell extracts from indicated cultures were prepared by glass beads beating in trichloroacetic acid, then resolved by SDS‐PAGE as previously described (Ren et al., 2016). The primary antibodies used in this study were anti‐Myc (9E10, monoclonal mouse hybridoma supernatant).

### 2.4 Calcofluor white staining and fluorescence microscopy

For cell wall observation, the indicated cultures were harvested and fixed with 70% ethanol. The fixed cells were washed and stained with a specific chitin stain calcofluor white 0.1% (Sigma‐Aldrich) for 15 min at room temperature. Images were taken using a Delta Vision Elite microscope (Applied Precision Inc., Mississauga, ON, Canada) with Volocity software.

## 3 RESULTS AND DISCUSSION

### 3.1 Overexpression of Cwp1 causes cell wall division defects and lethal

**Figure 1:**
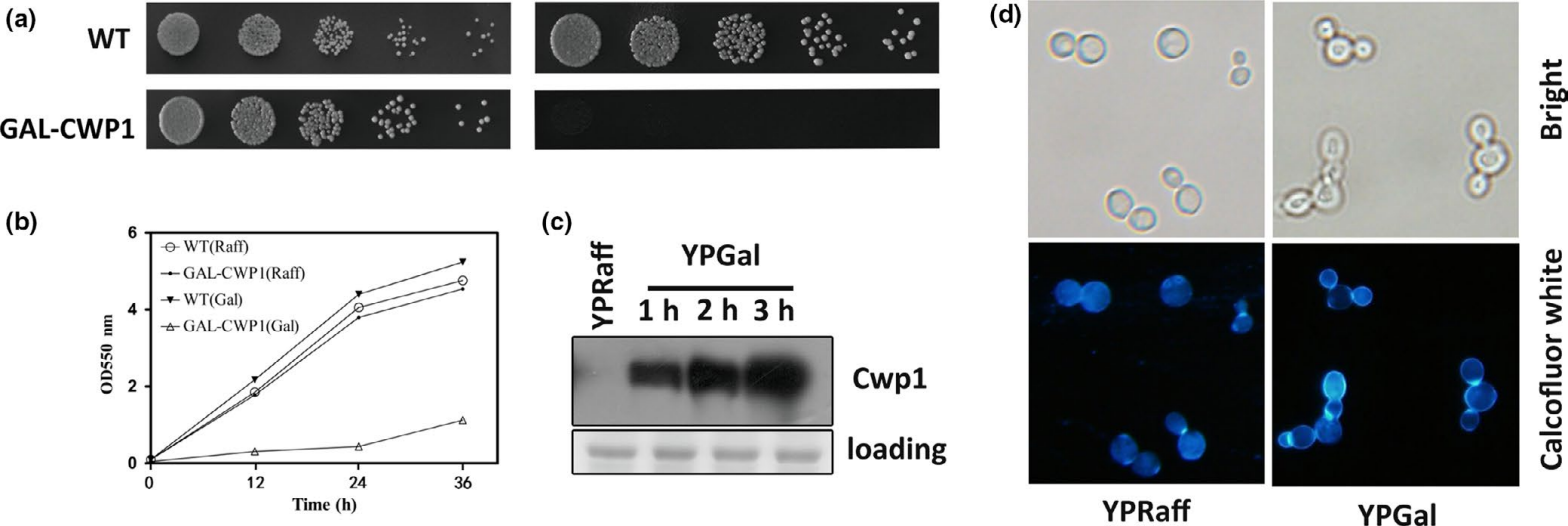
Inducible overexpression of Cwp1 leads to cell wall division defects and growth inhibition. (a) Fivefold serial dilutions of exponentially growing WT cells (YFL3) and Gal‐Cwp1 (YYH5) grown in YPRaff were spotted on YPD or YPGal plates at 30°C for 3 days. (b) WT cells and Gal‐Cwp1 with equal starting number were grown at YPRaff or YPGal and collected at indicated time for measuring the OD 550 nm. These data for the graph of the growth curves were the mean of three replicates. (c) Whole cell extracts analyzed by immunoblot with anti‐Myc (Cwp1) antibodies show that the proteins are being over‐expressed equally in the indicated strains. A Coomassie Blue‐stained region of the same membrane used for immunoblot is shown as a loading control. (d) Microscopy pictures showing cell morphology and Calcofluor staining of cell wall at identical scale (20 μm bar at right bottom corner).

Cell wall proteins are good candidates to be counter selection markers in yeast. Because induced overexpression of such proteins is promising to block yeast division due to cell wall division defects without cytotoxicity. We therefore screened a collection of cell wall proteins and found out Cwp1 as a good target. As shown in Figure 1a, overexpression of Cwp1 induced by galactose results in cell lethal. Same result was obtained by tracking cell growth in liquid culture (Figure 1b). Western blotting with anti‐Myc (Cwp1) antibodies shows that Cwp1 accumulates and keeps stable (Figure 1c). As expected, the robust cell growth arrest phenotype caused by Cwp1 overexpression results from cell wall division defects. Calcofluor white as a specific dye of cell wall component chitin was used to label the cell wall. As shown in Figure 1d, galactose addition significantly blocks cell division, leaving mother cells with multiple buds. Interestingly, cell wall division defects by Cwp1 overexpression could not block cytokinesis, suggesting that no cell cycle checkpoints or cytotoxicity was triggered. In addition, no obvious spontaneous recovery mutation was observed in Cwp1 overexpression system. Therefore, Cwp1‐induced cell wall division defects could be promising for counter selection.

### 3.2 Use of inducible lethal effect of Cwp1 for seamless gene deletion and marker recycling

**Figure 2:**
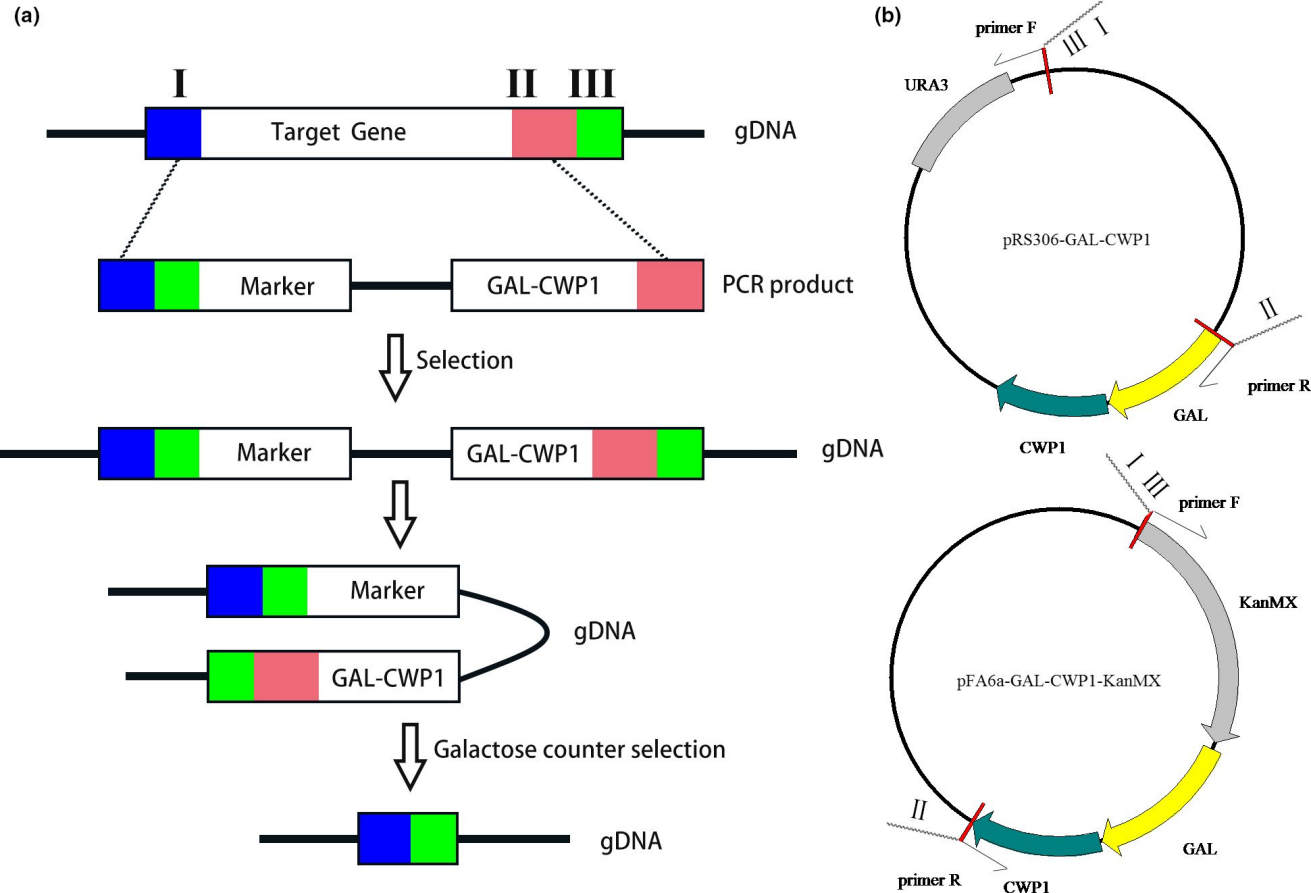
Outline of Cwp1 overexpression mediated seamless deletion method. (a) Cassette design for targeted gene deletion and seamless marker removal. Three sequences (>40 bp) adjacent to the targeted gene are chosen for cassette design. Sequences I and II are adjacent to the targeted gene, upstream, and downstream, respectively. Sequences III is downstream and adjacent to II. PCR products of the designed cassette using primers and templates as shown in panel A were then transformed. First‐round selection was done to get the strain with targeted gene replaced by GAL‐CWP1 cassette taking advantage of the selection marker included in the cassette. Galactose was then added and used to counter select the cells without GAl‐CWP1. (b) Cartoons depicting the structures of the indicated pFA6a‐based and pRS306‐based constructs for primers design to PCR up the GAL‐CWP1 and marker cassettes. The primers contain two fragment sequences including the 5′ end sequence annealing to the template constructs and 3′ end sequence (I, II, and III) designed as the homologous arm to the targeted gene

To make Cwp1 overexpression system attractive for yeast genetic manipulation, we took advantage of a strategy that applies homologous recombination between repeat sequences flanking a counter‐selectable Cwp1 cassette for its excision in the genome. The proposed methodology is described as shown in Figure 2a.

Three short sequences are selected as the homologous recombination arms in the following two rounds of homologous recombination events. In the yeast *S. cerevisiae*, homologous recombination arms with around 50 bp in size are efficient enough for gene targeting. As marked in the scheme, selected fragments I, II, and III are three 55 bp sequences at two sides of the gene or sequence to delete. Fragment I is a 55 bp sequence upstream the target to delete, fragment II is the last 55 bp sequence of the target, and fragment III is another 55 bp downstream sequence of the target.

The selected three sequences designed with oligo nucleatides complementary to template DNA for PCR annealing are synthesized as long primers. To make PCR easier, we constructed vectors pRS306‐GAL‐CWP1 and pFA6a‐GAL‐CWP1‐KanMX as the templates (Figure 2b). The primers are used to amplify the GAL‐CWP1 and selection marker cassette. PCR products from template of pRS306‐GAL‐CWP1 contain GAL‐CWP1:URA3 flanked with fragments I and III at the 5' terminus and II at the 3' end. Similar cassette with a drug‐resistant selection marker KanMX can be generated from pFA6a‐GAL‐CWP1‐KanMX template. The PCR products are transformed into the host cells and can replace the target sequence once homologous recombination takes place. After selection, mutant cells with targeted locus replaced with GAL‐CWP1 and selection marker are obtained.

Because the designed primers contain repeated flanking sequences, fragment replacing the targeted locus could be lost at a certain frequency, due to homologous recombination between repeated sequences on two sides. To select the mutant cells losing extraneous sequence, galactose is added to overexpress Cwp1 and make GAL‐CWP1 containing cells lethal. Therefore, the viable colonies are the favorite deletion mutants without any extraneous sequence at the targeted locus.

The proposed methodology for seamless gene deletion relies on two rounds of the homologous recombination and GAL‐CWP1‐dependent counter selection. GAL‐CWP1 and selection marker therefore can be used in the continued genetic editing in the same strain. In all, this strategy makes GAL‐CWP1 a nice seamless gene deletion and marker recycling system.

### 3.3 Efficient seamless deletion of ADE8 by GAL‐CWP1 system

To evaluate the proposed methodology, we chose ADE8 gene for this proof‐of‐principle experiment because the phenotype caused by ADE8 deletion can be visually screened. To delete ADE8 gene with GAL‐CWP1 system, we amplified the GAL‐CWP1:URA3 cassette from pRS306‐GAL‐CWP1 constructs using the primer pairs oYX7 and oYX8. Yeast cells w3031a YFL3 (WT) were transformed with PCR products of GAL‐CWP1:URA3 cassette and selected on the synthetic media lacking uracil. Positive colonies with ADE8 replaced by GAL‐CWP1:URA3 were white.

**Figure 3:**
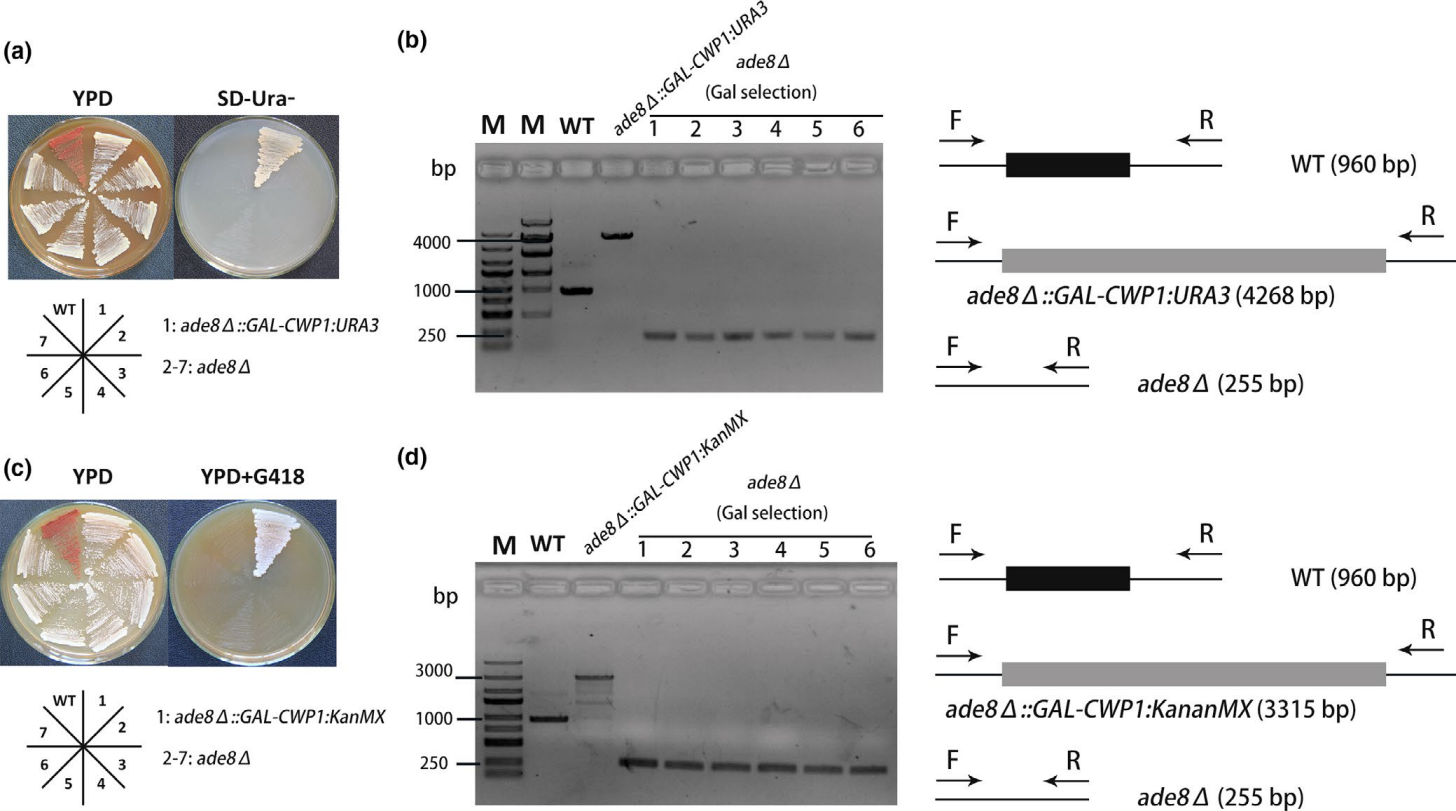
Analysis of the disruption of *Saccharomyces cerevisiae* ADE8 gene. (a, c) Functional analysis of *ade8* deletion mutants. Section 1 is a positive strain after first‐round selection containing Gal‐CWP1:URA3(KanMX) (URA3 in [a] and KanMX in [c]) at ADE8 loci. Sections 2–7 are six randomly picked colonies after Gal counter selection. (b, d) PCR analysis to confirm correct replacement of ADE8 by GAL‐CWP1 containing cassette and the removal at the ADE8 loci. M stands for DNA marker. Right lanes after marker are PCR products using gDNA from the indicated cells. WT: the starting wild‐type strain. *ade8Δ::GAL‐CWP1:URA3 (ade8Δ::GAL‐CWP1:KanMX)*: the indicated positive strain after first‐round selection containing Gal‐CWP1:URA3(KanMX) (URA3 in B and KanMX in D) at ADE8 loci. 1‐6:6 randomly picked colonies after Gal counter selection

Mutants were then grown in YPD media for 6 hr, followed by galactose counter selection on YPGal plates. Colonies grown on the YPGal plates were randomly picked for following confirmation. First, we checked the growth of the indicated mutant cells on SD‐Ura^‐^ plate. WT cells and all galactose selection colonies cannot grow on media lacking uracil (SD‐Ura^‐^) (Figure 3a), suggesting an efficient loss of GAL‐CWP1:URA3 fragment upon galactose selection.

To confirm the efficiency of seamless deletion of ADE8 by GAL‐CWP1 system, correct fragment integration was checked using a pair of primers with sequences complementary to the two sides of ADE8. As shown in Figure 3b, WT control template produced a ~1 kb PCR products band, *ade8* deletion mutants with ADE8 replaced by GAL‐CWP1:URA3 produced a ~4 kb band. One hundred percentage of the colonies after galactose counter selection gave ~250 bp bands which confirmed a high efficiency of ADE8 seamless deletion in this system. These short PCR products were confirmed by sequencing, further confirming the scarless deletion. This trial of deletion of ADE8 proved the proposed methodology using GAL‐CWP1 system for seamless deletion.

As for most industry strains, the use of auxotrophic selection is difficult because they are typically aneuploidy or polyploidy (Querol & Bond, 2009). To expand the usage of this method for industry strains, we further repeated the evaluation using KanMX as the dominant selection marker instead of auxotrophic selection. As shown in Figure 3c,d, 100% of the colonies turns seamless *ade8* deletion mutants using the proposed method.

To confirm the universality of GAL‐CWP1 system for seamless deletion, we have taken advantage of this method and efficiently generated other gene deletion strains. Genes deleted using this method in our laboratory include SML1*,* a small gene with a 208 bp open reading frame size; MEC1*,* a large gene with ~7 kb in size and among others (will be published elsewhere). Therefore, this method is feasible and supposed to be universal for any gene deletion in yeast.

GAl‐CWP1 system tends to be an ideal method for seamless gene deletion and marker recycling because of the following merits. First, CWP1 gene is derived from yeast genome and Cwp1 protein localizes to cell wall. Overexpression of Cwp1 leads to cell wall division defects but not block cytokinesis. The lethal effect is very strong and no significant spontaneous recovery mutants were detected. These features make GAL‐CWP1 a safe counter selection system. Second, the lethal effect of Cwp1 overexpression can be fast and simply achieved by galactose addition. The erase of GAL‐CWP1:marker sequence relies on short repeated homologous recombination arms which are designed in the primers.

In all, our results prove that cassette containing GAL‐CWP1 and a selection marker with repeated flanking sequences can be used for yeast genomic editing without any scar left behind in the genome. 55 bp homologous sequences are long enough to ensure the efficiency of the two rounds homology recombination in this method and therefore make GAl‐CWP1 system an efficient method for scarless gene deletion and marker recycling. Besides, cell wall protein genes are evolutionarily conserved among fungal species and the lethal effect might be also similar; therefore, this strategy could be used in other fungal species.

## 4 CONCLUDING REMARKS

In this study, we found that inducible overexpression of Cwp1 by galactose addition resulted in strong lethal effect. We evaluated that direct repeats flanking the Gal‐CWP1:selectable marker cassette allow for its homology recombination excision and counter selection upon galactose addition, therefore enable seamless gene editing and marker recycling. Our results highlight the utility of lethal effect of Cwp1 overexpression a new counter selection strategy and a simple and efficient method for seamless gene editing and marker recycling in *S. cerevisiae* and potentially other fungi.

## CONFLICT OF INTEREST

The authors declare no financial or commercial conflict of interest.

## AUTHORS CONTRIBUTION

FZ, ZH, and JD conceived the project and contributed to analysis and interpretation of data. FZ and YH participated in draft preparation and wrote the manuscript. YH, YJ, XZ, and ZY performed experiments. YH and YJ prepared the figures and wrote materials and methods section. All authors discussed and proofread the work and manuscript.

## ETHICS STATEMENT

We state that the ethics approval was not needed for this study, we still submitted our work to Ethics Committee in Hebei Agricultural University confirming no ethics issue related to our work.

## Data Availability

All data from the manuscript are deposited in FigShare (https://figshare.com/articles/_/7091060, https://figshare.com/articles/_/7091063, https://figshare.com/articles/_/7091066).
